# Spatial and Temporal Mapping of RF Exposure in an Urban Core Using Exposimeter and GIS

**DOI:** 10.3390/s25051301

**Published:** 2025-02-20

**Authors:** Montaña Rufo-Pérez, Alicia Antolín-Salazar, Jesús M. Paniagua-Sánchez, Antonio Jiménez-Barco, Francisco J. Rodríguez-Hernández

**Affiliations:** 1 Department of Applied Physics, School of Technology, Universidad de Extremadura, Avenida de la Universidad s/n, 10003 Cáceres, Spain; mmrufo@unex.es (M.R.-P.); paniagua@unex.es (J.M.P.-S.); 2 School of Technology, Universidad de Extremadura, Avenida de la Universidad s/n, 10003 Cáceres, Spain; asalazar@unex.es (A.A.-S.);

**Keywords:** personal exposimeters, radiofrequency electromagnetic fields, exposure quotiens, mobile measurements, outdoor multiple frequency environments, GIS

## Abstract

The primary aim of this study was to evaluate the spatial and temporal variation in human exposure to electromagnetic fields across different frequency bands within an urban area identified as the commercial zone of a medium-sized city. Central to this investigation was the use of an exposimeter, strategically positioned on the back of the operator and secured to the hip area via a belt, to ensure comprehensive and accurate field measurements. An initial analysis was conducted to determine the shielding coefficients of the human body, allowing for precise corrections of the electric field values used in the spatial assessment. To map power density across the study area for each frequency, kriging interpolation was applied. Furthermore, temporal variations in exposure levels were analyzed at three distinct times of day—morning business hours, afternoon business hours, and non-business hours—using robust statistical methods. The study’s innovative approach lies in the integration of GIS technology to uncover and visualize temporal patterns in exposure, particularly during periods of higher pedestrian density. This integration facilitated both the detection of temporal variations and the spatial representation of these changes, enabling rapid identification and assessment of exposure hotspots.

## 1. Introduction

The significant advancements in Information and Communication Technology have led to increased human exposure to non-ionizing radiation. This escalation has raised concerns both within scientific circles and society at large regarding the potential adverse effects of this exposure on human health. As reliance on technologies such as mobile phones, Wi-Fi networks, and other wireless devices continues to grow, so does the duration and intensity of exposure to electromagnetic fields. Consequently, there is a pressing need for comprehensive research to understand the health implications of prolonged exposure to non-ionizing radiation and to develop effective mitigation strategies to safeguard public health in this digital era. Various regulatory bodies worldwide have established exposure limits and guidelines to ensure the safety of individuals exposed to such fields, such as the International Commission on Non-Ionizing Radiation Protection [1,2,3], Federal Communications Commission (FCC) [4], Institute of Electrical and Electronics Engineers [5], as well as those established by the European Union in its recommendations, and in some cases, with limits set in regulations adopted individually by specific countries [6]. These guidelines serve as crucial references for setting occupational and environmental safety standards. Regular monitoring and measurement of electromagnetic fields help ensure compliance with these regulations and provide crucial information for epidemiological research. They also contribute to safeguarding public health and minimizing potential risks associated with prolonged exposure. By adhering to these exposure limits and continuously monitoring electromagnetic fields, we can mitigate potential health hazards and promote a safer environment for everyone. Amid concerns regarding exposure to non-ionizing radiation, the scientific community responds by conducting research to assess the levels to which the general public is exposed in their daily lives. Thus, there are studies where exposure levels have been determined in different microenvironments using various techniques, such as spectrum analyzers [7,8], exposure meters [9,10,11], or broadband meters [12,13].

The representation of electromagnetic field levels through Geographic Information Systems (GIS) plays a crucial role in understanding and managing human exposure to these radiations. GIS enables the spatially explicit visualization of electromagnetic field distribution in the environment, facilitating the identification of areas with elevated exposure levels and the evaluation of potential temporal and spatial variability patterns. This spatial representation is essential for urban planning, decision-making in public health policies, and risk communication to the population. Furthermore, GIS facilitates the integration of geospatial data with other relevant information types, such as demographic or environmental data, allowing for a more comprehensive and contextualized analysis of the potential impacts of electromagnetic fields on human health and the environment. There are many works that are using GIS to represent the exposure levels of non-ionizing radiation both indoors [14], and outdoors [8,12,15]. Geographic Information Systems (GIS) have emerged as indispensable tools in environmental research, particularly in analyzing spatial variations in environmental variables. The levels of exposure to non-ionizing radiation undergo temporal variations due to various factors, such as the implementation of new technologies in urban centers, such as 5G, the increase in base station antennas to expand urban coverage with this technology, temporal variations due to changes in population density in an urban area, changes resulting from scattering or reflection caused by buildings. These temporal variations have been studied by different authors through statistical analyses [16,17,18]. Thus, Sánchez-Montero et al. [12] observed broadband variations over a 5-year period between 2006 and 2010, with smaller variations recorded for the period between 2010 and 2016. Similarly, other researchers, such as Fernández et al. [16], analyzed the temporal variation in Wi-Fi exposure levels in the 2.4 GHz band within a university setting, employing statistical methods. However, the use of GIS technology can be very useful in this type of study, as it can not only determine the existence of these temporal changes but also establish the degree of change in different study areas. This way, areas where a study of these levels over time would be necessary could be selected.

The main objectives of our work have been twofold. Firstly, to conduct a spatial study of exposure levels in an urban area characterized as the commercial zone of a medium-sized city. Through the use of an exposimeter, we determined exposure levels resulting from mobile telephony and FM emissions, which are predominant in the urban area. Secondly, we conducted a temporal study of these exposure levels at three different times of the day using statistical analysis, distinguishing between morning business hours, afternoon business hours, and non-business hours. However, the primary and most innovative objective was to utilize GIS technology to determine the presence or absence of temporal variations in exposure levels at different times of the day characterized by potentially higher pedestrian traffic in the area. This enabled us to verify the existence or absence of temporal variations in the study area and classify such variations into three distinct categories based on their magnitude: irrelevant variations, moderate variations, and high variations. This method can be applied to any spatially represented magnitude, providing relevant information on areas where frequent temporal studies would be necessary.

## 2. Materials and Methods

### 2.1. Environment

**Figure 1:**
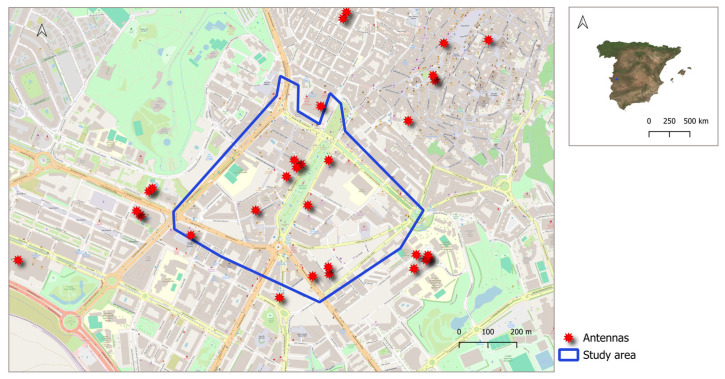
Digital map of the study area in Cáceres with the mobile telephony antennas positions.

The study was conducted in the commercial district of a medium-sized city located in the west of Spain, specifically in Cáceres, which has an approximate population of 96,117 inhabitants according to the 2021 census. The study area covered an area of 0.37 km^2^ and is notable for being the busiest commercial zone within the city. The population density in the study zone varies according to the schedule, reaching maximums during business hours. In the area where this study was performed, there were 25 mobile telephony antenna sites, in many cases shared by different technologies. Of these, 12 operated in the 800 MHz band, 28 in the 900 MHz band, 21 in the 1800 MHz band, 17 in the 2100 MHz band and 3 in the 2600 MHz. Their locations on the city map, obtained from data provided by the Ministry of Energy, Tourism, and the Digital Agenda [19] are shown in Figure 1. The FM and Television antennas are located on the outskirts of the city, specifically there are five locations with different antennas in the frequency band from 87.5 to 108 MHz located to the south, north and west of the city [20].

### 2.2. Instrumentation

The data collection of the measurements of the electric field strength in different points of the area was carried out with a personal exposure meter (PEM) EME spy 200 (Microwave Vision Group, Courtaboeuf, France) [21]. This device is a selective, isotropic and portable electromagnetic field meter with 20 pre-defined frequency bands in the frequency range of 87–5850 MHz. The frequency range includes (FM) radio broadcasting, (TV) television broadcasting, (TETRA) Terrestrial Trunked Radio, (GSM) global system for mobile communications, (UMTS) universal mobile telecommunication, (LTE) Long term evolution, (DEC) digital enhanced cordless telecommunication, (Wi-Fi) wireless local area networks, and (WiMAX) worldwide interoperability for microwave access. The dynamic range is 61.6 dB (up 6 V/m) and the measurements uncertainties (Axial isotropy Vertical) are listed in Table 1. The meter can store 80,000 data points measuring with a recording interval from 4 to 255 s and has the ability to geo-locate the measurements with GPS position. The technical characteristics of the PEM are listed in Table 1, including the frequency band, the sensitivity, and the axial isotropy.

The use of exposimeters is well-established in research for their ability to rapidly and efficiently collect data across large areas [9,10,11,15,22]. These devices enable continuous and geo-referenced measurements, making them particularly suitable for studies requiring high spatial and temporal resolution in diverse environments. Additionally, exposimeters are designed for straightforward deployment without the need for further adjustments, enhancing their usability in field studies.

**Figure 2:**
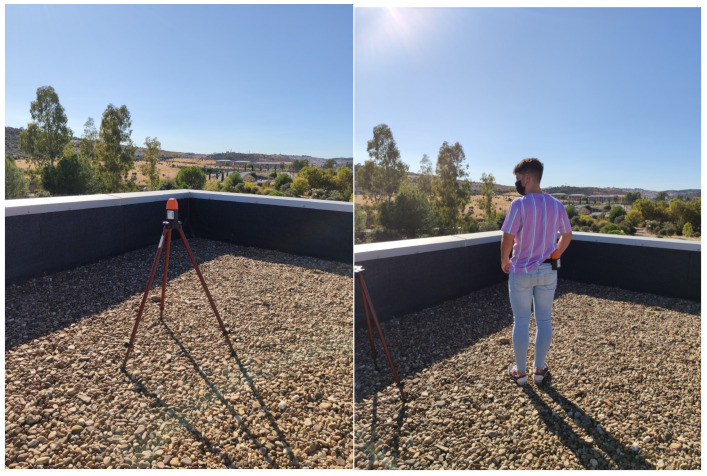
PEM located at the waist and on a tripod, taking the measurements corresponding to the study of shielding of the human body.

The measurements were made walking along the different selected streets of the city center, always at a constant speed. The same measurements were taken three times at three different moments: morning business hours, afternoon business hours, and non-business hours. The exposure meter was placed on the back of the operator, attached to the belt in the hip area. As is known, the operator’s body can produce positive and negative alterations in the reception of the magnitude of the electric field [9]. Thus, a measurement protocol was previously carried out to estimate the shielding of the operator’s body in the measurement process. The objective was to obtain average shielding coefficients for the different frequencies, which would be used to correct the data obtained in the study of the commercial area. For this purpose, a measurement point was chosen where two types of measurements were made. One of which was carried out on a tripod for 2 min at 4 s intervals. At the same point, eight measurements were taken with the exposimeter attached to a belt in the hip area and turning 45° clockwise starting with the north orientation. Measurements were made in each orientation for 2 min at 4 s intervals (see Figure 2).

### 2.3. Data Processing

Any device used to determine electric field levels in the environment has detection limits (DL) that are sometimes not exceeded, resulting in what is known as censored data. Various methodologies have been utilized and examined by different authors to deal with this type of data [23,24,25,26]. In our case, we have considered frequency bands with higher presence in urban areas, such as those corresponding to mobile phone and FM. In this instance, the number of censored data points is well below 50% and has been replaced by DL/2. This is a straightforward method that does not compromise precision given the low percentages of censored data obtained in the selected bands in our study. The exposure assessment in our study has been conducted through two procedures. Firstly, the values of power density have been determined for each of the frequency bands under investigation. On the other hand, according to the ICNIRP [3], the criterion for exposure to multiple-frequency sources to prevent thermal effects should take into account the electric and magnetic fields and power density. For practical application of the whole-body average reference levels, incident electric field strength, incident magnetic field strength, and incident power density values should be added according to Equation (1):

(1) $$\begin{array}{c} Q_{Th}\\ =\sum_{i=100\;kHz}^{30\;MHz}\left\{{\left(\frac{E_{inc,i}}{E_{inc,\;RL,i}}\right)}^{2}+{\left(\frac{H_{inc,i}}{H_{inc,\;RL,i}}\right)}^{2}\right\}\\ +\sum_{i>30\;MHz}^{2\;GHz}MAX\left\{{\left(\frac{E_{inc,i}}{E_{inc,\;RL,i}}\right)}^{2},\;{\left(\frac{H_{inc,i}}{H_{inc,\;RL,i}}\right)}^{2},\left(\frac{S_{inc,i}}{S_{inc,RL,i}}\right)\right\}\\ +\sum_{i>2\;GHz}^{300\;GHz}\frac{S_{inc,i}}{S_{inc,RL,i}}\leq 1 \end{array}$$

where *E_inc,i_* and *E_inc,RL,i_* are the local incident electric field strength and local incident electric field strength reference level for local exposure [3], at frequency *i*, respectively; *H_inc,i_* and *H_inc,RL,i_* are the local incident magnetic field strength and local incident magnetic field strength reference level for local exposure [3], at frequency *i*, respectively; and *S_inc,i_* and *S_inc,RL,i_* are the local incident power density and local incident power density reference level for local exposure, frequency *i*, respectively.

### 2.4. Spatial Analysis

The electric field strength values were interpolated using an interpolation method to represent the values spatially. The primary objective was to obtain a spatial representation of exposure levels to easily identify areas with lower and higher electric field values for different frequencies bands. A total of 557 points were analyzed.

Interpolation was carried out using ordinary kriging [7], chosen as the method that best fits the data, assuming that the constant mean value is unknown and there is no trend in the data. For its calculation, a logarithmic transformation was established since the data do not follow a normal curve. The free program utilized for its calculation was SAGA version: 7.9.1 [27], as it allows for the study and modification of the empirical variogram that best fits the dataset. Thus, the calculation has predefined the function that best fits, varying the range to the correlation distance and the point cloud.

Following this methodology individually for each type of schedule and for each frequency band studied, ordinary kriging with electric field strength values was achieved, thus obtaining one kriging for each schedule (morning business hours, afternoon business hours, and non-business hours). Similarly, standard deviation maps for each of them are also calculated.

To study the temporal variation in electric field strength values and thus establish the areas of greatest variation between different schedules, map algebra is performed to find the difference between them, pairwise. That is, the difference between the values of the morning business hours and afternoon business hours, morning business hours and non-business hours, and afternoon business hours and non- business hours. This step is carried out using the “Raster Calculator” present in the QGIS program version 3.16.11 Hannover [28], producing three raster maps that show these variations, which must be studied in their absolute value, a process that is carried out again with the raster calculator.

Once the absolute value difference maps are obtained, the maps are reclassified into three values. Due to the low variability of the data between different schedules, a decision was made to carry out the reclassification based on the division into tertiles (three data classes). A value of 0 will be given to the values contained in the first tertile, 1 to those in the second tertile, and 2 to those in the third tertile. The intervals can be chosen as desired by the operator, either by tertiles or by selected fixed values, providing flexibility in the classification approach.

According to these reclassifications, value 0 would correspond to pixels exhibiting minimal variation between the two schedules, value 1 to represent average difference or variability, and value 2 for areas or pixels displaying greater oscillation. The same procedure would be applied to the three different maps, resulting in three reclassified maps with values 0, 1, and 2, based on the degree of difference between data from different schedules. The final map delineating the varying zones among the three schedules was derived from the summation of the three reclassified rasters. Consequently, the final output will be a raster with values ranging from 0, indicating minimal variation, to 6, representing maximum variation. These high-value pixels are areas where all three difference maps between schedules have registered a value of 2, indicating the greatest discrepancy. Once the three maps are added, a resulting map with pixels with values 0, 1, 2, 3, 4, 5, and 6 will be obtained. This final cartography will be represented using a symbology of three intervals ranging from 0 to 1 for the minimum variation zone between schedules, 2–4 for medium variability, and 5–6 for those areas with maximum oscillation between schedules. These intervals can be modified based on the values of the dataset.

## 3. Results and Discussion

Firstly, we conducted a study on the percentage of values exceeding the limit of detection of the PEM. Thus, Table 2 presents the limit of detection for each frequency band and the percentage of values exceeding that limit for each band detected in the study area. As can be observed in Table 2, the highest percentages detected correspond to the FM band and mobile telephony band DL. It is worth noting that for the GSM + UMTS 900 DL, GSM 1800 DL, and UMTS 2100 DL frequency bands, we obtain 100% detectable values. For the FM, LTE 800 DL, and DECT bands, the percentage of censored values is very small: 0.2%, 0.2%, and 4.2%, respectively. However, for the LTE 2600 DL band, the values exceeding DL decrease slightly (74%) compared to other mobile telephony DL bands. Among the bands with less representation in the study area, we can highlight the Wi-Fi 2G and Wi-Fi 5G bands, for which we obtain a percentage of detectable values of 26.8% and 29.2%, respectively.

Based on the data shown in Table 2, we focus our study on the downtown area of the city, specifically on the most representative frequency bands: the FM band and mobile telephony bands, including LTE 800 DL, GSM + UMTS 900 DL, GSM 1800 DL, UMTS 2100 DL, and LTE 2600 DL.

### 3.1. Shielding Factors of Human Body

**Figure 3:**
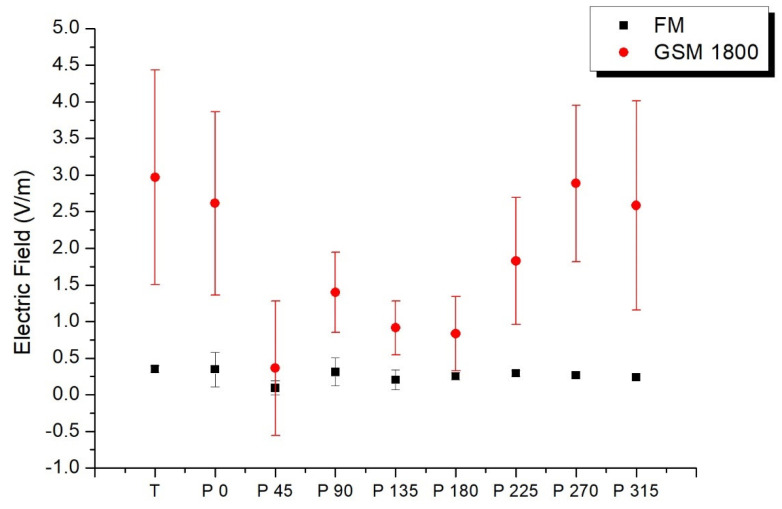
Average value of the electric field strength (V/m) from the 30 measurements taken on the tripod (T) and at each orientation, Pα.

In order to obtain shielding coefficients of the human body, measurements were conducted using the PEM positioned at the hip and varying the orientation of the exposure meter every 45 degrees at a location in the University of Extremadura. Figure 3 depicts a plot of the electric field alongside the standard deviation obtained for each orientation, as well as the values obtained with the PEM positioned on the tripod, for two of the six emissions considered in our study: FM and GSM 1800 (DL). This figure presents the average value of the electric field strength (V/m) from the 30 measurements taken on the tripod (T) and at each orientation α, with 0 degrees aligning with the north direction. For the three emissions, variations are observed between the values obtained on the tripod and those obtained with the PEM in different orientations. The lowest values in both cases are obtained at the 45-degree orientation, while the highest values are obtained when the PEM is not shielded by the human body; in such cases, signal reception is even higher than that obtained with the exposure meter measuring on the tripod.

Table 3 presents the average values of the shielding coefficients E_α_/E_T_ for the emissions examined in the study, along with the mean electric field values for each frequency band and their corresponding standard deviations in V/m and %. The highest electric field values are observed in the two GSM bands, attributed to the proximity of a cellphone tower to the measurement point. In contrast, lower mean values are recorded for emissions from the LTE 2600 and UMTS 2100 bands, as antennas for these frequencies are situated at a greater distance. The shielding coefficients range from 0.49 to 0.71, with the lowest value observed for the FM band and the highest for LTE 2600. Notably, shielding coefficient values for the cellphone bands exhibit relative similarity.

The values obtained in the spatial study were corrected with the mean shielding coefficient values for each frequency band studied.

### 3.2. Spatial Interpolation

Table 4 presents a statistical summary of the total power density in the study area during different sampling times: morning, afternoon, and non-commercial hours. It is worth noting that the highest values detected occurred during the morning commercial hours and were slightly lower during the afternoon commercial hours. However, based on the data displayed in Table 4, there does not appear to be a significant difference in representative values, such as the mean or median, across the different time periods.

**Figure 4:**
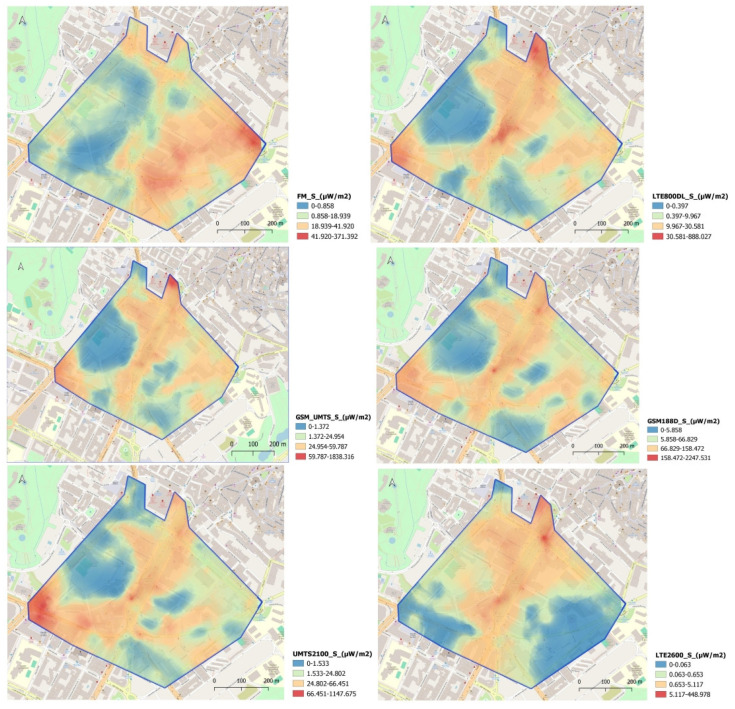
Interpolated map depicting the power density (μW/m^2^) for various frequency bands using the kriging method.

Figure 4 depicts the spatial representation of the FM power density and that corresponding to different emissions of mobile telephony DL for the sampling conducted during the business morning hours. The method utilized is ordinary kriging, which has been proven to be optimal for such environmental variables in complex scenarios such as cities [7,29]. The figure illustrates how the power density obtained for the GSM 1800 DL band produces the highest exposure values in the study area. Additionally, the spatial distribution of power density differs significantly between FM and mobile telephony bands in the study area. The highest values for the FM band are located in the western part of the city, whereas for mobile telephony they are observed in the central area, where an avenue is located, which has less influence from buildings.

However, for mobile telephony emissions, a very similar spatial distribution among bands is observed. The variation in the distribution of the different values obtained for the various frequency bands justifies representing each of them separately. This representation allows us to observe the spatial differences between the two types of emissions analyzed. This phenomenon may be influenced by the fact that different emission bands share antenna locations, leading to a similar distribution of electric field intensity levels. Thus, Table 5 presents a correlation study conducted for the electric field value detected in the study area for each of the studied emissions. As we can observe, the correlation coefficients are significant consistently for the electric field values between mobile telephony and DL frequency band, while significant coefficients are not obtained in the correlation between mobile telephony and FM values. Notably, coefficients of 0.8 for UMTS 2100 DL and GSM 1800 DL, or 0.7 for correlations between GSM + UMTS 900 (DL) and LTE 800 DL, are observed. This fact is also reflected in the spatial representation of power density for each of the mobile telephony emissions in the study area, where a significant similarity is observed in the spatial distribution of values for those emissions in which we find higher coefficients of significant correlation.

**Figure 5:**
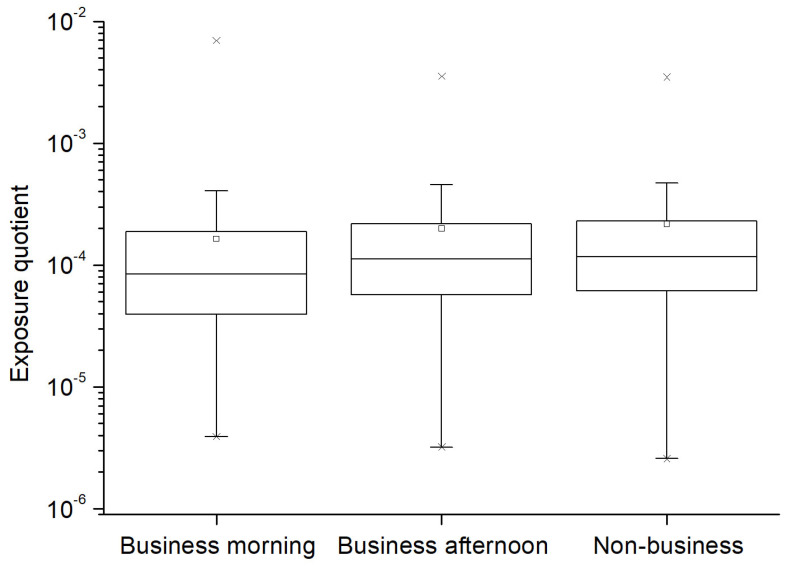
Box-and-whisker plot of the exposure quotients Qth in business morning, afternoon, and non-business hours.

Furthermore, it is worth noting that the exposure levels do not exceed the limits established by regulatory guidelines in the study area. Thus, Figure 5 presents a box-and-whisker plot of exposure quotients for different time intervals. The maximum values obtained in the spatial analysis of these coefficients are 0.00697 during the morning business hours, with values of 0.00354 and 0.00348 during the afternoon business hours and non-commercial hours, respectively. Supplementary Material S1 (Figure S1) presents the spatial distribution of thermal stimulation quotient Q_Th_ in the study area during the morning commercial hours. It is noteworthy that the values are well below the limit set at 1. Additionally, we observe that the area of minimal radiation exposure is the western zone of the study area, which remains in a blue hue. Exposure increases in this case in the western part of the influenced area due to the contribution of the predominant FM band in this zone.

### 3.3. Temporal Study

In Table 6, a statistical summary of the electric field values obtained in the study area for different sampling times is presented: business morning hours, business afternoon hours, and non-business hours. The table details the values by frequency bands, including the minimum and maximum values, mean, median, standard deviation, percentiles, range, and interquartile range. Observing the values, it appears that the ones sensitive to sampling time are the maximum values. We find higher maximum values during non-business hours for the FM band, LTE 800, and GSM + UMTS 900. However, for the GSM 1800 and UMTS 2100 bands, the highest maximum values are obtained during business morning hours. For the LTE 2600 band, the highest maximum value is detected during non-business hours.

Looking at the 95th percentile value for the electric field, we can see that in this case, higher values are obtained in all bands during business morning hours. The standard deviation values are elevated, indicating a considerable spatial variation in electric field values in the study area. For the FM band, the values are nearly 70%, while for the mobile telephony bands, they are higher in all cases, ranging between 85% and over 100%.

**Figure 6:**
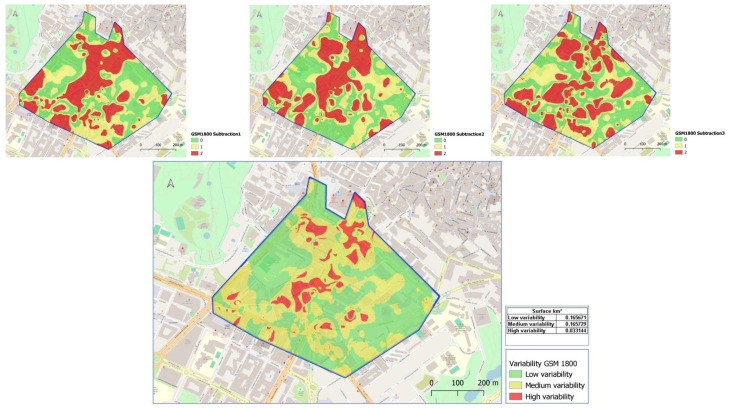
Reclassified maps and the final map for GSM 1800 frequency band, derived from the summation of the previously reclassified raster.

The temporal variability of power density in the area has been studied using a geographic information system. As an example, in Figure 6, the reclassified maps with differences in power density for different pairwise time intervals are presented at the top for the GSM 1800 (DL) frequency. The bottom figure shows the sum of the three previous maps, allowing us to visually, quickly, and easily identify areas of higher temporal variability among the three time intervals studied. In this case, a numerical value for the variability is not obtained; instead, we show through cartography the areas with high, medium, and low temporal variability. Thus, areas of high variability can be quickly identified visually. Using GIS tools, the surface area of each zone with different variability has been determined. Therefore, we can observe in Figure 6 the surface area obtained for each studied range. This result allows us to, on the one hand, consider the importance of this temporal variability and, on the other hand, identify those areas within the study area that should be considered for further temporal frequency analysis.

As seen in the final map, for the GSM 1800 (DL) frequency, the area of greatest temporal variability corresponds to a wide pedestrian zone, free of buildings, where emissions do not seem to be shielded. It is an area to be considered, as it corresponds to a zone with a higher pedestrian flow, which is a sensitive part of the city. It can be stated that the area considered to have high temporal variability within the study zone is not very extensive, accounting for 9.2% of the total area considered. According to the results obtained, it can be affirmed that the surface area with high temporal variation is low considering the time intervals considered in this study. The area of low and medium variability is similar in percentage, approximately 46% of the total area for each.

Figure S2 presents the final cartography for the remaining frequency bands studied in the research. Among them, it is noteworthy that for FM emissions, we obtain the minimum area of the zone with high temporal variability, in this case accounting for 4.8% of the total study area. The frequency band exhibiting the largest area of temporal variability corresponds to the LTE 2600 frequency band. In this instance, the high variability surface area is 15%. It is worth noting that this frequency band records the lowest exposure values. In the case of FM frequency, the area of highest variability does not coincide with those of mobile telephony bands. Instead, the high variability zone is small and situated in the western part of the study area map. However, it does coincide with the area of highest exposure for that frequency.

## 4. Conclusions

In this study, we have assessed electromagnetic field exposure in a medium-sized city’s commercial area. The primary objective has been to employ geographic information systems to represent both spatial and temporal variation. In the spatial representation of power density, similarities are observed among the frequency bands used for mobile telephony, while this similarity is not present with the FM band. Exposure in the commercial area largely meets the criteria established for multiple frequency sources, yielding coefficient values lower than 0.00697, the maximum value obtained for the morning commercial hours. Spatial variations in the study area are greater than the temporal variations observed at three different times of the day: morning, afternoon, and non-commercial hours. Variations due to increases or decreases in pedestrian density in the area do not seem to be noticeable. However, an optimal methodology has been developed to represent and quantify, through surface calculation, the temporal variation in the electromagnetic field in a city or part thereof using GIS technology. This approach not only provides quantification of temporal variation ranges through statistical tools but also offers a map representation of areas showing greater temporal variations, visually limiting the area for possible subsequent studies.

## Figures and Tables

**Table 1:** Technical characteristics of the personal exposure meter, frequency band, sensitivity (DL), and axial isotropy.

Frequency Band	Frequency Range (MHz)	Sensitivity E (mV/m)	Axial Isotropy Vertical (±dB)
FM	87–107	10	0.1
TV3	174–223	10	0.5
TETRA I	380–400	10	0.2
TETRA II	410–430	10	0.5
TETRA III	450–470	10	0.5
TV4&5	479–770	10	0.3
LTE 800 DL	791–821	5	0.3
LTE 800 UL	832–862	5	0.3
GSM + UMTS 900 UL	880–915	5	0.3
GSM + UMTS 900 DL	925–960	5	1.0
GSM 1800 UL	1710–1785	5	1.2
GSM 1800 DL	1805–1880	5	1.2
DECT	1880–1900	5	0.9
UMTS 2100 UL	1920–1980	5	0.6
UMTS 2100 DL	2110–2170	5	0.8
Wi-Fi 2G	2400–2483.5	5	0.6
LTE 2600 UL	2500–2570	5	0.9
LTE 2600 DL	2620–2690	5	0.9
WiMAX	3300–3900	5	2.2
Wi-Fi 5G	5150–5850	10	2.2

**Table 2:** Frequency bands, sensitivity, and percentage of measurements that exceeded the PEM’s limit of detection (DL).

Frequency Band	Sensitivity DL (V/m)	% Measurement > DL
FM	0.010	99.8
TV3	0.010	0.2
TETRA I	0.010	2.7
TETRA II	0.010	0.1
TETRA III	0.010	0.3
TV4&5	0.005	67.6
LTE 800 DL	0.005	99.8
LTE 800 UL	0.005	15.1
GSM + UMTS 900 UL	0.005	2.6
GSM + UMTS 900 DL	0.005	100
GSM 1800 UL	0.005	57.8
GSM 1800 DL	0.005	100
DECT	0.005	95.8
UMTS 2100 UL	0.005	5.1
UMTS 2100 DL	0.005	100
Wi-Fi 2G	0.005	26.8
LTE 2600 UL	0.005	1.1
LTE 2600 DL	0.005	73.9
WiMAX	0.005	0.0
Wi-Fi 5G	0.010	29.2

**Table 3:** Mean electric field values (E), standard deviation (σ), and average shielding coefficient values (Q = E_α_/E_T_) obtained for each frequency band.

	FM	LTE 800 (DL)	GSM + UMTS 900 (DL)	GSM 1800 (DL)	UMTS 2100 (DL)	LTE 2600 (DL)
E (V/m)	0.25	0.56	1.2	1.7	0.045	0.04
s (V/m)	0.08	0.26	0.6	0.9	0.024	0.04
s (dB)	0.67	2.0	4.1	5.6	0.21	0.34
s (%)	30.8	46.6	49.4	56.5	54.3	94.1
Q = E_α_/E_T_	0,71	0.63	0.68	0.57	0.50	0.49

**Table 4:** Statistical summaries of power density (μW/m^2^) measured at three different moments of a day. IQ—interquartile; R—range.

mW/m^2^	Business Morning	Business Afternoon	Non-Business
Savg	978	1231	1194
s	2900	2331	1903
Smin	18.8	14.2	10.3
S25	179	256	261
S50	434	543	498
S75	1034	1283	1171
S95	3051	4513	4622
Smax	60,856	31,987	13,453
IQ	855	1027	910
R	60,837	31,973	13,443

**Table 5:** Linear correlation coefficients between electric field strength for different bands’ frequencies. Values in boldface are statistically significant (*p* < 0.05).

LTE 800 DL	GSM + UMTS 900 (DL)	GSM 1800 (DL)	UMTS 2100 (DL)	LTE 2600 (DL)	
0.021	−0.016	0.012	−0.039	−0.005	FM
	**0.696**	**0.631**	**0.484**	**0.537**	LTE 800 (DL)
		**0.559**	**0.491**	**0.527**	GSM + UMTS 900 (DL)
			**0.771**	**0.557**	GSM 1800 (DL)
				0.354	UMTS 2100 (DL)

**Table 6:** Statistical summaries of the electric field (V/m) measured in three different moments of a day: BM (business morning hours), BA (business afternoon hours) and NB (Non-business hours). IQ—interquartile; R—range.

		E_avg_	σ	E_min_	E_25_	E_50_	E_75_	E_95_	E_max_	IQ	R
FM	BM	0.134	0.105	0.010	0.059	0.111	0.176	0.522	0.896	0.118	0.889
	BA	0.165	0.110	0.010	0.087	0.141	0.217	0.350	0.773	0.130	0.762
	NB	0.190	0.131	0.010	0.108	0.164	0.247	0.392	1.644	0.139	1.633
LTE 800	BM	0.147	0.155	0.008	0.049	0.098	0.176	0.849	1.128	0.127	1.120
	BA	0.148	0.146	0.008	0.055	0.101	0.189	0.425	1.011	0.133	1.003
	NB	0.161	0.168	0.008	0.062	0.105	0.203	0.498	1.428	0.141	1.420
GSM + UMTS 900	BM	0.181	0.148	0.019	0.084	0.132	0.225	0.697	0.944	0.142	0.925
	BA	0.176	0.144	0.016	0.078	0.129	0.224	0.524	0.775	0.146	0.758
	NB	0.183	0.170	0.019	0.079	0.129	0.225	0.504	1.394	0.146	1.375
GSM 1800	BM	0.275	0.235	0.034	0.131	0.219	0.345	1.025	3.494	0.214	3.460
	BA	0.331	0.253	0.035	0.161	0.262	0.419	0.798	2.073	0.258	2.037
	NB	0.311	0.254	0.039	0.136	0.227	0.386	0.915	1.952	0.250	1.913
UMTS 2100	BM	0.194	0.189	0.020	0.082	0.141	0.251	0.819	2.743	0.169	2.723
	BA	0.226	0.219	0.018	0.098	0.173	0.277	0.556	2.713	0.179	2.695
	NB	0.195	0.160	0.020	0.086	0.149	0.257	0.508	1.251	0.171	1.231
LTE 2600	BM	0.089	0.165	0.005	0.005	0.030	0.095	0.818	1.763	0.090	1.758
	BA	0.111	0.213	0.005	0.012	0.045	0.132	0.415	2.668	0.119	2.663
	NB	0.118	0.203	0.005	0.005	0.049	0.130	0.516	1.852	0.124	1.847

## Data Availability

The datasets used and/or analyzed during the current study are available from the corresponding author on reasonable request.

## Supplementary Materials

The following supporting information can be downloaded at: https://www.mdpi.com/article/10.3390/s25051301/s1, Figure S1: Interpolated map depicting exposure quotients Q_Th_ using the kriging method; Figure S2: Final maps of variability for frequency FM, LTE 800 DL, GSM + UMTS 900 DL, UMTS 2100 DL, and LTE 2600 DL.
