# Supplementary material for: Spatial and Temporal Mapping of RF Exposure in an Urban Core Using Exposimeter and GIS
Source: Sensors (Basel). 2025 Feb 20;25(5):1301. doi: 10.3390/s25051301 (PMC11902647; doi:10.3390/s25051301)
Supplement: Supplementary file 1 [file sensors-25-01301-s001.zip › sensors-3439735-supplementary.pdf]

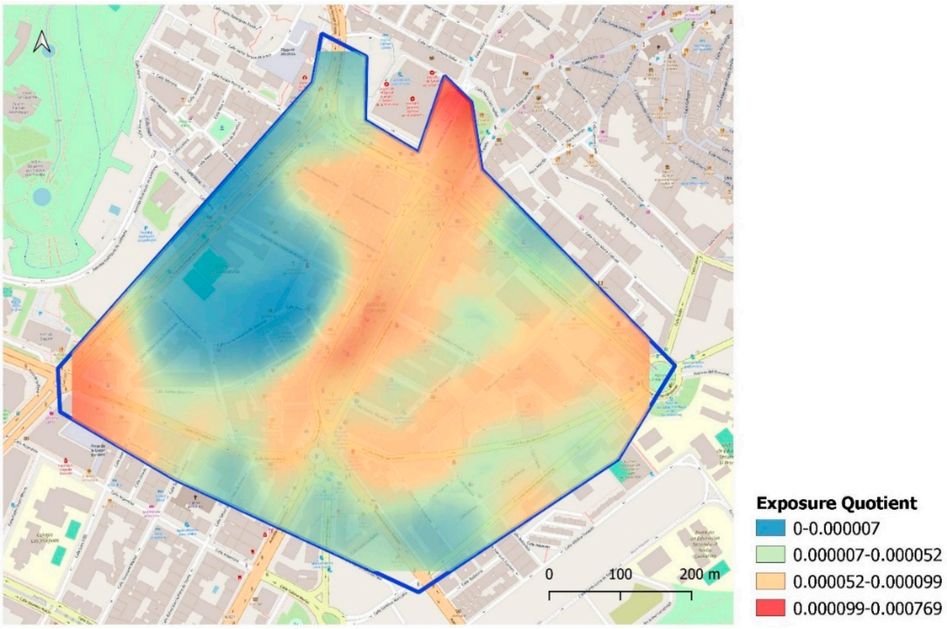

Figure S1. Interpolated map depicting exposure quotients QTh using the kriging method.

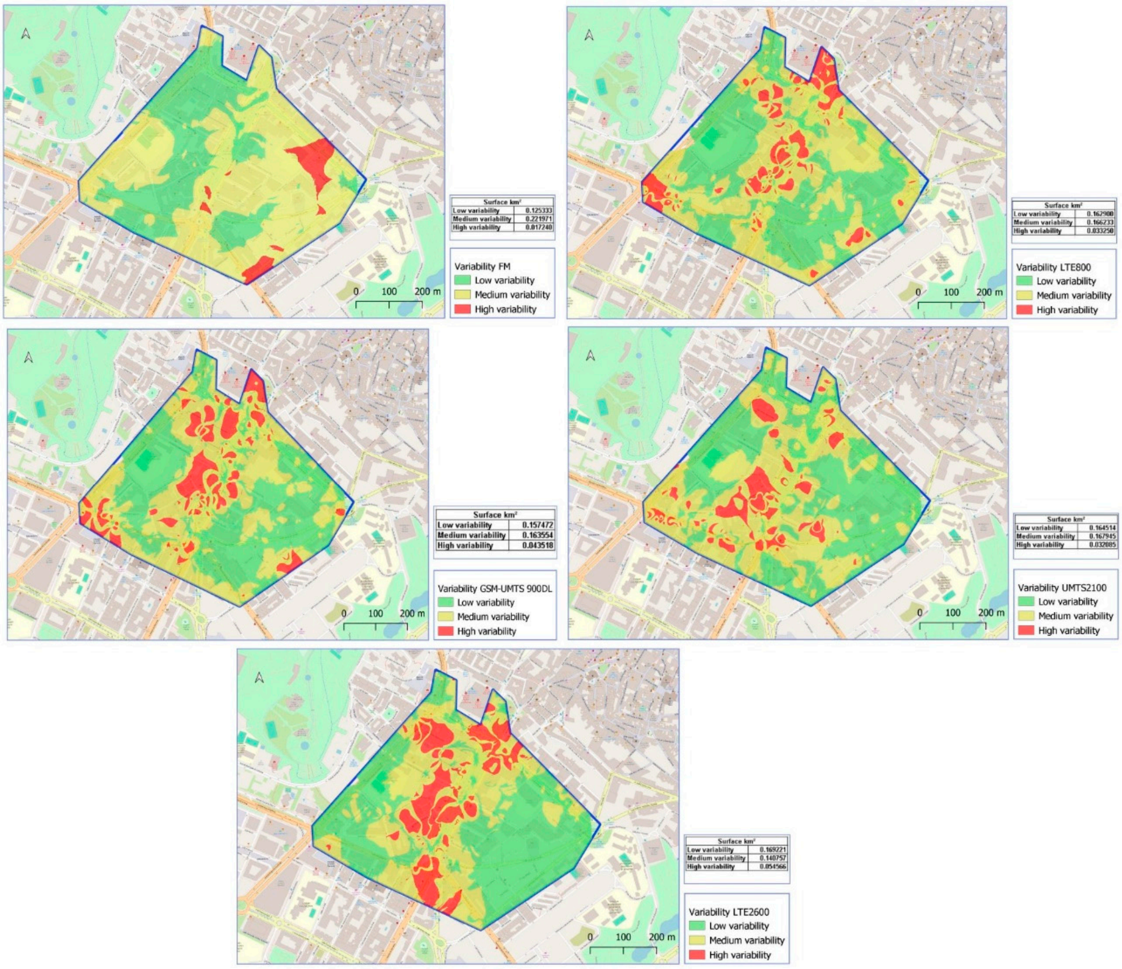

Figure S2. Final maps of variability for frequency FM, LTE 800 DL, GSM + UMTS 900 DL, UMTS 2100 DL, and LTE 2600 DL.
